# Multi-source evaluation of an educational program aimed at medical students for interviewing/taking the clinical history using standardized patients

**DOI:** 10.3205/zma001436

**Published:** 2021-02-15

**Authors:** Sophia Denizon Arranz, José Manuel Blanco Canseco, María Montserrat Pouplana Malagarriga, María Soledad Holgado Catalán, María Isabel Gámez Cabero, Antonio Ruiz Sánchez, Diana Monge Martín, Roger Ruiz Moral, Santiago Álvarez Montero

**Affiliations:** 1 Francisco de Vitoria University, Faculty of Health Sciences, School of Medicine, Madrid, Spain

**Keywords:** educational program, standardized patients, clinical history, medical students

## Abstract

**Introduction: **Simulations with standardized patients (SP) have long been used for teaching/assessing communication skills. The present study describes and evaluates an experiential training methodology aimed at medical students and based on interviews with standardized simulated patients. The training was focused on developing basic communication skills and taking medical histories.

**Methods:** Longitudinal observational study of a cohort of third-year medical students. Three interviews with SP were carried out and videotaped. These interviews were assessed by the students, the SPs and the professors of the relevant subject areas.

**Results: **83 students conducted the interviews. The self-evaluations performed by the students showed an improvement between the first and third interviews, as demonstrated by the increase of 6.7% (CI 95%=3.6-10.0%) (p<0.001) in the percentage of detected items. The SPs stated an improvement of 8.5% (CI 95%=2.9-14.1) (p=0.003) from the first to the third interview regarding the percentage of students that showed a level of interest in, and ease with, the patients’ concerns. Finally, the teachers found a mean percentage of items identified in the third written clinical history of 61.4% (CI 95%=59.1-63.7) of the total available.

**Conclusions: **This educational program, carried out with standardized simulated patients, showed positive signs of improvement from the first to the third interview, in both the student self-evaluations and the level of interest and ease perceived by the SPs. Additionally, the mean level of information recorded in the written medical histories was considered to be acceptable.

## Zusammenfassung

**Einführung: **Simulationen mit standardisierten Patienten (SP) werden seit langem für die Unterricht/Bewertung von Kommunikationsfähigkeiten eingesetzt. Die vorliegende Studie beschreibt und evaluiert eine erfahrungsbasierte Trainingsmethodik, die sich an Medizinstudenten richtet und auf Interviews mit standardisierten simulierten Patienten basiert. Das Training konzentrierte sich auf die Entwicklung grundlegender Kommunikationsfähigkeiten und die Erfassung von Krankengeschichten.

**Methoden:** Längsschnittbeobachtungsstudie an einer Kohorte von Medizinstudenten im dritten Studienjahr. Es wurden drei Interviews mit SP durchgeführt und auf Video aufgenommen. Diese Interviews wurden von den Studierenden, den SPen und den Professoren der entsprechenden Fachbereiche ausgewertet.

**Ergebnisse:** 83 Studenten führten die Interviews durch. Die von den Studierenden durchgeführten Selbsteinschätzungen zeigten eine Verbesserung zwischen dem ersten und dem dritten Interview, wie der Anstieg des Prozentsatzes der entdeckten Items um 6,7% (CI 95%=3,6-10,0%) (p<0,001) zeigt. Die SPen gaben eine Verbesserung von 8,5% (CI 95%=2,9-14,1) (p=0,003) zwischen dem ersten und dem dritten Interview an, was den Prozentsatz der Studierenden betrifft, die ein gewisses Interesse an den Bedenken der Patienten zeigten und mit diesen leicht umgehen konnten. Schließlich fanden die Lehrer einen mittleren Prozentsatz von 61,4% (CI 95%=59,1-63,7) der insgesamt verfügbaren Items, die in der dritten schriftlichen Krankengeschichte identifiziert wurden.

**Schlussfolgerungen:** Dieses Bildungsprogramm, das mit standardisierten simulierten Patienten durchgeführt wurde, zeigte positive Anzeichen einer Verbesserung vom ersten bis zum dritten Interview, sowohl in den Selbsteinschätzungen der Schüler/innen als auch im Grad des Interesses und der Leichtigkeit, die von den SPen wahrgenommen wurde. Zusätzlich wurde das mittlere Informationsniveau, das in den schriftlichen Krankengeschichten aufgezeichnet wurde, als akzeptabel angesehen.

## 1. Introduction

The clinical interview is probably the most frequently performed activity in clinical practice [1]. Therefore, eliciting a good clinical history (CH) from a patient is an essential element of effective medical intervention [2]. History taking consists of obtaining relevant personal, psychosocial and symptomatic information from the patient in order to acquire useful data for making a diagnosis and providing medical care [3].

The classic study of Hampton, published in 1975, showed that 66-80% of patients were correctly diagnosed by the CH alone [4]. Other studies show that the CH provides from the majority of new patients on internal or general medicine clinical settings enough information for making a diagnosis even before any physical examination or complementary tests [5]. 

Taking a good CH requires establishing a set of communication guidelines aimed at building a clinical relationship that facilities the collection of relevant information and stablishes a positive therapeutic partnership from the first moment of contact.

Educational programs aimed at improving communication skills have been developed in the majority of medical schools in the United States [6], Canada [7], Germany [8] and the United Kingdom [9] and Spain [10] where they are considered essential. National accreditation agencies and various expert consensuses have highlighted the importance of training programs that focus on the fundamental skill of taking a CH [11], [12], [13]. 

It is well known that experiential pedagogic methods are the most effective [14], [15], [16]. However, training of this kind continues to be a challenge, with important questions left unresolved. For example, how can this type of methodology be implemented while producing the least amount of “suffering” for the students [9], [17], [18], [19]? Furthermore, when and in what way should it be implemented [17], [20]? What concrete strategies should be applied in changing educational environments, and with which groups of students [21], [22], [23], [24], [25]? 

It has been a challenge to introduce training for the CH interview for students with no previous experience of rotations in health centers in order to to start off their internships as efficiently as possible.

Along the last years, our medical school has developed a curriculum for learning interpersonal skills whose main characteristics are: its development throughout the whole curriculum (from the first to the last year), with diverse contents (from basic communication skills to more specific topics, such as managing emotions, breaking bad news or motivational interviewing) and with experiential methodologies: teaching in small groups, simulated patient interviews, structured feedback and rehearsals, encouraging group discussions and the student's personal reflection on their performance. Details about different aspects of this program and its impact have been published elsewhere [26], [27], [28], [29], [30].

Simulation with SPs has proven to be useful, both as an instruction tool and to assess communication and clinical skills, such as investigative and reasoning skills [31]. In addition, it leads to an improvement in patient safety by reducing the number of potential mistakes in real clinical practice [32]. The SP training is the best way to guarantee the authenticity and reproducibility of clinical simulated encounters [33], [34], [35]. Students have greatly value this experience [36], [37].

Authenticity and feedback have been highlighted as two of the most important variables for determining the quality and the SP training [30]. Our SP receive a training program in which the priority is to guarantee both factors, through a high and diverse pool of SP (authenticity) and a specific scheduled training that includes feedback on their performances and on the way in which the SP themselves must provide feedback to students [38]. When interviews with SPs are recorded, the feedback of second-year medical students is more positive than when these interviews are only observed [39]. 

The evidence in favor of using SPs in communication skills training for taking CH is growing [40], [41], [42], [43], but still a bit scarce in specific areas [3]. Although there is enough broadly evidence that these skills can be taught, studies with standardized patients and workshops with small groups of preclinical students are sparse. In the Keifenheim et al. systematic review [3] only 6 studies were carried out with SPs, and of them, only two assessed specifically history taking [37], [44]. We provide additional evidence supporting their results by using and assessing an experiential methodology aimed at third-year medical students. The training was based on interviews with SPs to teach basic communication skills (including the perceived level of comfort and interest of students during the clinical interview) and how to take the CH. 

Its objective is to determine if this training is effective in helping students achieve these skills prior to the beginning of their clinical practice and to know about the learner perception about the experience. To make it, we conducted a longitudinal observational study of a cohort of medical students. We registered student self-evaluation of the videotaped interviews and the written history as well as the SPs’ assessment. In addition, we explored the teacher-student assessment correlation in the evaluation of the written history and considered the student opinion by a survey.

## 2. Methods

### 2.1. Design and scenario

The study was conducted as a longitudinal observational study of a cohort of medical students. It was undertaken during the “Clinical Methods I” (CM I) course in the fourth month of the third year of the medical degree curriculum. The main objective of CM I course consists on training students to establish an adequate clinical relationship during the clinical interview and teaching them how to gather significant clinical data through anamnesis and physical examination. 

The specific training undertaken during CM I mainly consists teaching how to get and record a traditional CH (anamnesis; "history taking") and how to do a basic physical examination (clinical semiology; the “physical exam”), as well as how to cover certain basic procedures to properly use some devices (for instance a peak-flow-meter). They also are initiated on some basic communication skills needed in order to develop a patient-focused interview. These basic skills consisted, on the one hand, on being careful respecting elementary courtesy rules (self-introduction to patients, making them feel comfortable, requesting consent to complete a medical record) and, on the other hand, getting information about the patients perspective (their idea about their health problem cause, their experience over the impact of their symptoms on their lifestyle, or their concerns and knowledge of the condition).

The subject (CM I) is taught for six weeks, and includes: 24 hours of theoretical classes with participative methodologies, 26 hours of practical workshops in a simulation environment, three interviews with SPs that simulate clinical scenarios with different pathologies, and three briefing seminars after the interviews. CM I is taught before students begin their rotations at health centers, allowing them to practice the skills obtained in training. In the fourth year of medical study, the CM II subject complements the knowledge acquired in CM I, and deepens training in communication skills.

#### 2.2. Participants

Among the 126 third-year medical students who were invited to participate in the program, 111 actively participated. There were nine teachers giving the course, all of whom were primary care physicians.

When the SPs had no previous experience, their training included a preliminary introduction session, two main sessions or specific training workshops, and one or more shorter complementary sessions with fewer objectives (Standardized Patient Training Program of the Francisco de Vitoria University Medical School in Madrid) [38].

Scripts for the SP roles were based on real clinical cases and were developed by two clinicians.

#### 2.3. Training intervention

**Figure 1:**
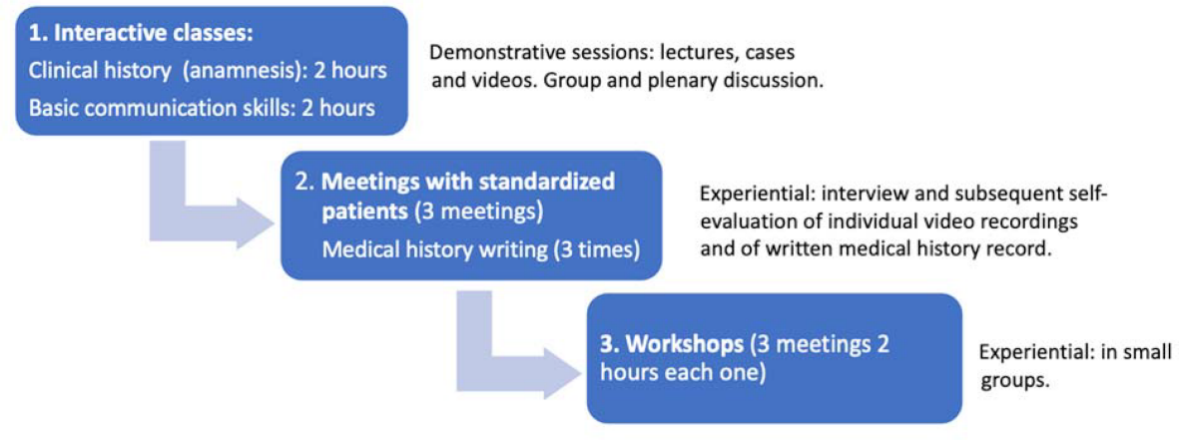
Sequence of teaching intervention

Along the training undertaken within CM I course every student received 28 hours of theorical classes and 22 hours of workshops to practice physical exams among themselves or with simulators when needed (for instance to recognize cardiac and pulmonary pathologic sounds), 3 interposed interviews with SP and 1 workshop after each SP encounter (3 in total) focused to feedback. There was no role-playing in small groups neither virtual or real patients in our training program. Our program was different from the traditional approaches (lectures or review of videos) because, in addition to these approaches, standardized patients were interposed. Afterwards, students worked in small group sessions where two facilitators collected their impressions and concerns about these encounters. The educational intervention, specifically aimed at improving the know-how of history taking and basic communication skills, had the following components (see figure 1 (Fig. 1)):

##### 2.3.1. Demonstrative sessions and sessions in small groups

During the first session, the CH anamnesis structure is discussed. The second session focuses on the basic communication skills needed for establishing a doctor-patient relationship, and for collecting and providing information. Through presentations, video clips and situations designed to prompt discussion, relevant information was given by the teacher to the students, encouraging the whole class to reflect. Later on, students work in small ad hoc groups that are created according to their shared skills and experience followed by an exchange. 

##### 2.3.2. Individual interviews with SPs

These meetings are carried out at the Advanced Clinical Simulation Center, which offers all the necessary resources for various simulation scenarios. The students perform three encounters with three different SPs. The clinical situations faced by students are carried out in a general medicine context. The SP scenarios consist of the following:

1. a 35-year-old female patient who comes to the office with pain in her right wrist as a consequence of a fall (eventual diagnosis: scaphoid fracture);
2. a 60-year-old male patient who has developed chest pain over the course of two months with the usual characteristics (eventual diagnosis: unstable angina);
3. a 35-year-old female patient who comes to the office expressing fatigue, having gradually lost weight over the last month (eventual diagnosis: onset of diabetes mellitus).

These encounters are performed sequentially during the symptomatology program. The clinical problems that arise in each encounter according to the subject matter that students were studied and received training on. Besides that, they were of increasing difficulty in terms of history taking skills.

Each encounter consists of a 15-minute clinical interview that is videotaped. The students are then given 15 minutes to write the CH (on paper).

After each encounter with the SP, the student watches their videotaped interview via the online system (the Learning Space Program). They then assess their performance using a specific self-evaluation template (see an example in table 1 (Tab. 1)), as well as their written CH using another self-evaluation template designed for this purpose (see table 2 (Tab. 2)). The checklist for the written CH is structured in four blocks:

1. Preliminaries and reason for consultation;
2. Personal history and lifestyle;
3. Family history; and
4. Current disease (see an example in table 2 (Tab. 2)).

Each student evaluates their own performance through the completion of this form. 

Teachers compiled the list of history taking skills/tasks and built a tailored checklist scale to assess the level displayed by the student in the interview. There were some little differences among checklists to fit them to each case training objectives required. This scale was discussed by the group of teachers and its final version was agreed (face validity). To collect the opinion of the SP, a simple grading scale was proposed, its levels were discussed with the simulated patients.

In order to evaluate the students’ opinion of this teaching program, they also complete a survey consisting of seven items (see table 3 (Tab. 3)). Each item is formulated as a statement and assessed on a six-point, single, ordinal, Likert-type scale: 1-2=less useful and comfortable; 3-4=intermediate score; 5-6=useful and comfortable.

The teachers had no role during the interviews except to keep aware that everything is going on in the right way. The feedback comes up from students’ auto-evaluation watching their own videos and written CH along with the checklist tool.

##### 2.3.3. Group workshops

Once the students have evaluated themselves, they then take part in a group workshop consisting of 30 students. The class is split into four or five smaller groups in order to discuss their experiences of the clinical encounter and CH. The workshop is conducted according to the established training principles for small groups, and is moderated by two teachers. The material addressed in this session is shown in table 4 (Tab. 4). The main measures of effect were: items correctly identified in viewing the first and third videotaped interviews, items correctly identified in the evaluation of the third written CH, correlation about teachers’ and students’ evaluation of the third written CH, standardized patient’s subjective opinion about basic communication skills of students (feeling of comfortableness along the interview and perception of student’s interest in patient’s viewpoint and experience), and the measures related to students’ opinion survey.

**Student-related measures (self-evaluation)**

- Percentage of items correctly identified in viewing the first and third videotaped interviews carried out by the student with the SP.
- Percentage of items correctly identified in the evaluation of the third written CH (using the checklist).
- Results of the opinion survey.

**SP-related:** Level of improvement of the student’s basic communication skills from the first to the third interview. After the interviewing, the SP should have to rating two sentences to express his/her own perception, from 1 (the worst) to 3 (the best):

1. The interviewer has made me feel comfortable (he/she has been kind and respectful).
2. The interviewer has shown interest in my concerns about my problem.

**Analysis of correlation between teachers’ and students’ evaluations:** An analysis of the correlation between the self-evaluation of the third written CH carried out by the students and the teachers’ evaluation as “clinic experts” is carried out in order to detect where the students most overvalue (or overestimate) their perception as compared to the values outlined by the teachers. The differences detected will be useful for implementing improvements in the next academic year.

#### 2.4. Ethical statement

All local, regional and national standards, laws and regulations have been respected. In addition, participants have filled an informed consent procedure for recording sessions, have voluntarily participated in all the procedures described below and the necessary steps to ensure anonymity in the management of the data have also been taken.

#### 2.5. Statistical analysis

Mean, median and standard deviation (SD) were used to analyze the percentage of correct items in each student’s self-evaluation of the CH and recorded interviews, and the percentage of occasions in each interview in which the student made the SP feel comfortable and attentively listened to. 

To analyze the differences, the mean percentage of the observed improvement in the detection of items between the first and the third interview was calculated together with the corresponding confidence interval (CI) of 95%. The paired Student’s t-test was used for comparing means.

To assess the differences between the percentage of items detected in the CH by the student, and the items detected by the “clinical expert” (the teacher), the percentage of occasions where the student overestimated their perception with regard to the teacher’s results was used. Four teachers participated in this assessment, and each of them evaluated a quarter of the clinical histories written by the students. Prior to this evaluation, the interobserver concordance index was analyzed by the Kappa coefficient for each item. A coefficient greater than 0.8 was achieved on all occasions. 

The analysis was carried out using IBM SPSS statistical software for Windows v.21, and the alpha level of significance of <0.05 was considered.

## 3. Results

### 3.1. Student self-evaluation of the videotaped interviews

Among the 111 students who participated in the program, 84 students conducted the three interviews. The mean percentage of items correctly identified by the students after viewing the videotapes increased from 73.6% in the first interview (median 76.2%, SD 11.6%) to 79% (median 80.7%, SD 13.2%) in the third interview. The mean variation of the improvement in the percentage of detected items was 6.7% (CI 95%, 3.6-10.0%) (p<0.001).

#### 3.2. Evaluation performed by the standardized patients (SP) 

The mean percentage of occasions in which the SP perceived that the student made him feel comfortable (being kind and respectful) and observed the student's interest in his concerns regarding his health problem was 73.6%73.6% in the first interview (median 75%, SD 28.4%) and 82.0% (median 75%, SD 19.7%) in the third interview. The mean difference was 8.5% (CI 95%, 2.9-14.1) (p=0.003).

#### 3.3. Evaluation of the third written CH: teacher and student correlations

**Figure 2:**
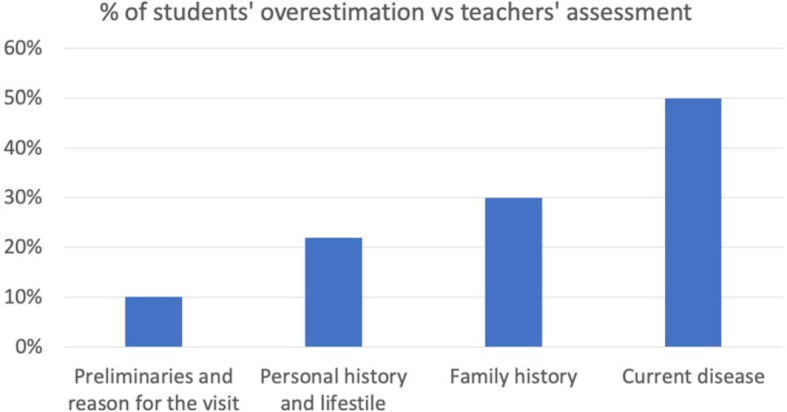
Teacher and student correlations. (Third clinical history). Overestimated student self-evaluation

Among the 71 students who completed the self-evaluation of the third CH, a mean of 70.6% (CI 95%=66.9-73.3%) of the total items was detected using the evaluation checklist. When the evaluation was performed by the teachers, this value was 61.4% (CI 95%, 59.1-63.7). By analyzing the results from the four blocks in greater detail, it was found that:

1. Preliminaries and reason for consultation: students overestimate 10% of the data with respect to the teachers’ external evaluation (see figure 2 (Fig. 2)).
2. Personal history and lifestyle: students overestimate 22% of the data with respect to the teachers’ external evaluation (see figure 2 (Fig. 2)).
3. Family history: students overestimate 30% of the data with respect to the teachers’ external evaluation (see figure 2 (Fig. 2)).
4. Current disease: students overestimate 50% of the data with respect to the teachers’ external evaluation (see figure 2 (Fig. 2)).

#### 3.4. Results of the student opinion survey 

**Figure 3:**
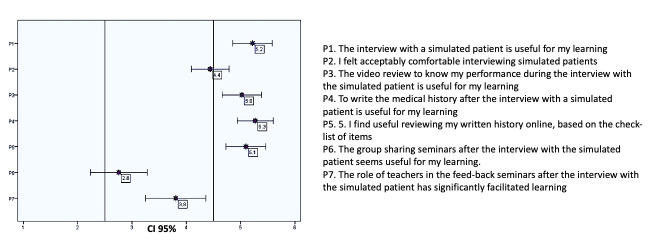
Student survey results (CI 95%)

The survey response rate was 36.9% (n=41). The highest scores (on a scale of 1 to 6) were detected in the assessment of the usefulness of the SP interview (mean 5.2 points), the self-evaluation of the videotaped interview using a checklist (mean 5.0 points), the task of writing the CH (mean 5.2 points) and the self-evaluation of the written history using a checklist (mean 5.0 points). The mean score related to feeling comfortable during the interview was 4.4 points. The lowest scores corresponded to the feedback seminars (mean 2.7 points) and the significant impact made by teachers’ interventions on the learning experience (mean 3.8 points) table 3 (Tab. 3), figure 3 (Fig. 3) shows the mean scores obtained for each item with CI 95%.

## 4. Discussion

Communication skills can be acquired and mastered by practice, and experiential learning is very important in this regard [45]. SPs are most commonly used for

1. teaching communication skills, and
2. teaching clinical skills [46].

Our results show an increase on percentage of detected items by student’s videotape assessment, an improvement of the SPs’ perception of students’ basic communication skills and a student’s survey with high scores about usefulness of SP interview and self-evaluation, but low scores on feedback and teacher’s intervention in seminars held after each interview. Most of these data are in accordance with those obtained in other studies [3] in which students and instructors showed high levels of satisfaction with SP methodology [47].

If we consider the possibility that SP perception improvement could be related to an improvement of students’ basic communication skills, then the simulation could be useful to prevent real patients’ unpleasant feelings or an impression that the medical professional does not show enough interest in their problems. 

Despite the limitations of our students’ self-evaluation found out in our correlation study of the written CH, they showed a tendency to improve that was not attributable to chance alone, as demonstrated by the mean percentage of items identified in the first and third videotaped interviews. This improvement coincides with the perceptions of the SPs, who discerned a significant improvement in concerns for their health from the first to the third interview. Actually, this may due to different causes such as habituation effect and not only to an improvement of students’ basic communication skills. Anyway, this result does not contradict the data obtained in published literature [14], [15], [16]. In addition, it demonstrates the progress that can be achieved with reflexive learning. Only two studies assessed preclinical students history taking through SP interventions [37], [44]. It was through self-evaluation questionnaire and course evaluation [37] and through OSCE and writing stations [44]. Our study intervention is similar but, additionally, we performed a multi-source evaluation, which includes self-assessment of students, their evaluation of the course, the evaluation by teachers of the written clinical history and the evaluation of the communication perceived by the SP.

When analyzing the correlation between the external evaluation by teachers and the self-evaluation by students after the written CH was completed, there were two results that were of particular pedagogical and evaluative interest:

1. the student self-evaluation was overestimated (excessively favorable), or
2. the external evaluation was either in line with the self-evaluation or the student was more rigorous than the teacher in the evaluation.

The first result is not desirable since it may suggest a low level of self-reflection. However, it may also suggest that we have to improve some items to rephrase them in a more specific and clearer way. Otherwise, students may tend to interpret ambiguous sentences in their own sake. When items could be interpreted differently, the percentage of overestimation may rise. If this one was so, we have to review, for instance, the items formulated in the "Family background" and "Current disease” sections.

The high percentage of items collected in the written CH by the students and verified by observers is likely attributable to students’ theoretical training, engagement with practical workshops and the first and second SP interviews. The results can be improved, but they are encouraging given the large amount of items that must be evaluated. Our study agrees with those by Von Lengerke et al. [37] and Battles et al. [44], in the fact that the standardized patient seems to be a key factor for improving their practical skills for history taking. 

Many studies have suggested that medical students suffer from high levels of stress and anxiety throughout their undergraduate training, and that these anxiety levels may influence the type of communication students establish with patient [47]. This finding would justify the score relating to levels of comfort with the experience (i.e. the interviews with the SPs) stated by the students in our study. Although this score was not particularly high, it seems acceptable, considering the high level of stress that this training methodology usually produces in students [9], [17], [18], [19]. Our study agrees with Von Lengerke et al. [37] about the student positive opinion on standardized patients as a training intervention. 

The negative attitudes expressed with regard to how students feel may signal that students have a negative perception of the way these skills are taught [17]. However, this does not necessarily correspond with negative attitudes toward the benefit of using such skills when seeing patients [17]. Indeed, the high score obtained in the statements related to the usefulness of interviews in simulation conditions demonstrated students’ satisfaction with the experience and the overall adequacy of the teaching. This result is significant and is in accordance with previous research [36], [47]. On the other hand, the low score obtained with regard to the group workshops suggests that these should be reviewed. The students answered survey open questions saying that the amount of time dedicated to each feedback seminar done after each SP interview was excessive. They found them "not very useful" because they were "repetitive" or "too long". Their feedback were very useful to manage future seminars.

The design of the study implies certain methodological limitations, as it was conducted as a longitudinal observation of a cohort of students without a control group. It has not been possible to make a comparison between the items recorded in the CH in the first and the third interviews, as the teaching objectives of the CH were different, being more extensive in the third than the first interview. The difference between the number of students enrolled in the course and those who completed the study is difficult to interpret. The difference between the students who completed the first and third interviews and those who recorded the CH in writing is 15%. These decreases in percentage are difficult to interpret with the data that is available, and are probably due in part to the non-summative nature of the evaluation. In any case, further research is needed regarding the impact of this type of educational intervention on the collection of relevant information by students. 

## 5. Conclusions

This educational program aimed at medical students for interviewing and taking clinical histories using SPs, showed positive signs of improvement from the first to the third interview. 

The student self-evaluations of the videotaped interviews using a checklist showed an increase of checklists collected items percentage. 

From first to third interview, there was an improvement of participating SP’s perception on their comfort levels and sensation of interest from students in their medical concerns.

The mean level of information recorded in the written medical histories has been considered to be acceptable given the increasing complexity of interviews, although improvable for future courses.

Scores of students’ perception about SP interviews and self-assessment shown that they found this experience useful and acceptably comfortable.

## Competing interests

The authors declare that they have no competing interests. 

## Figures and Tables

**Table 1:**
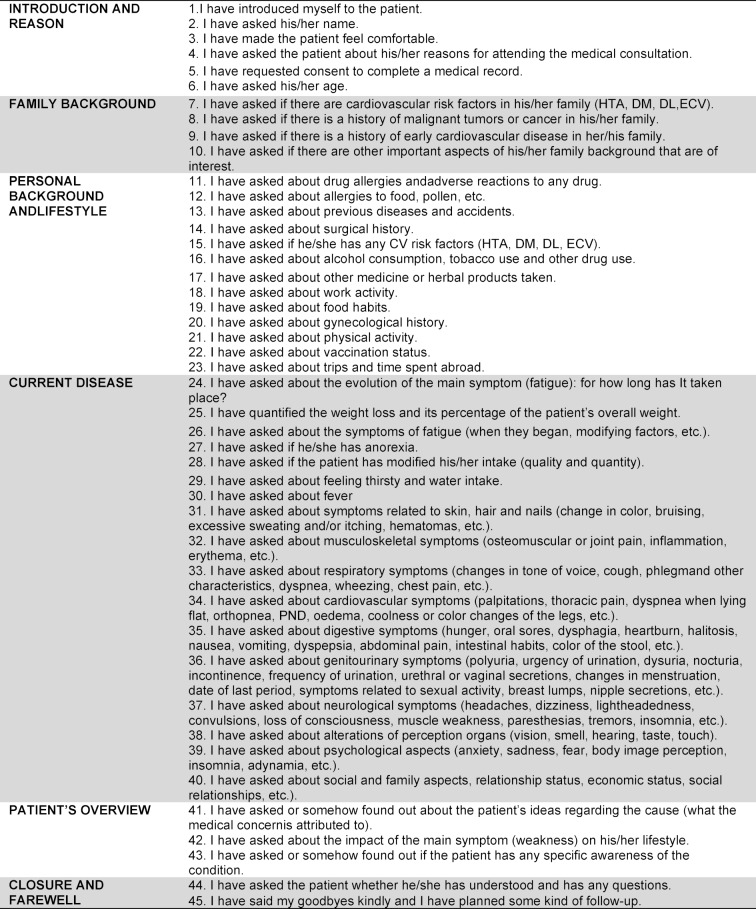
Items checklist for the self-evaluation of the videotaped interview

**Table 2:**
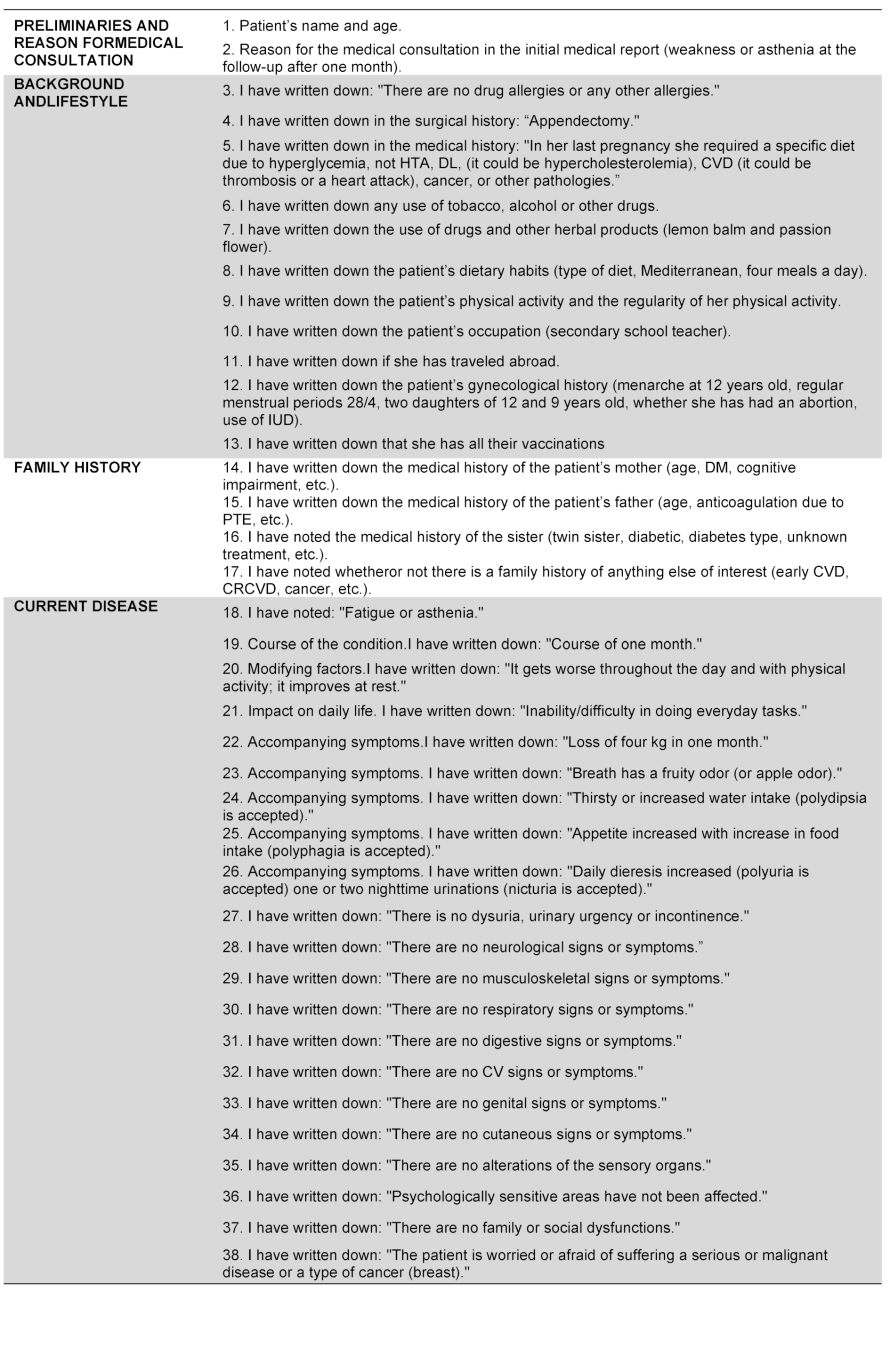
Checklist and items for the self-evaluation of the written medical history

**Table 3:**
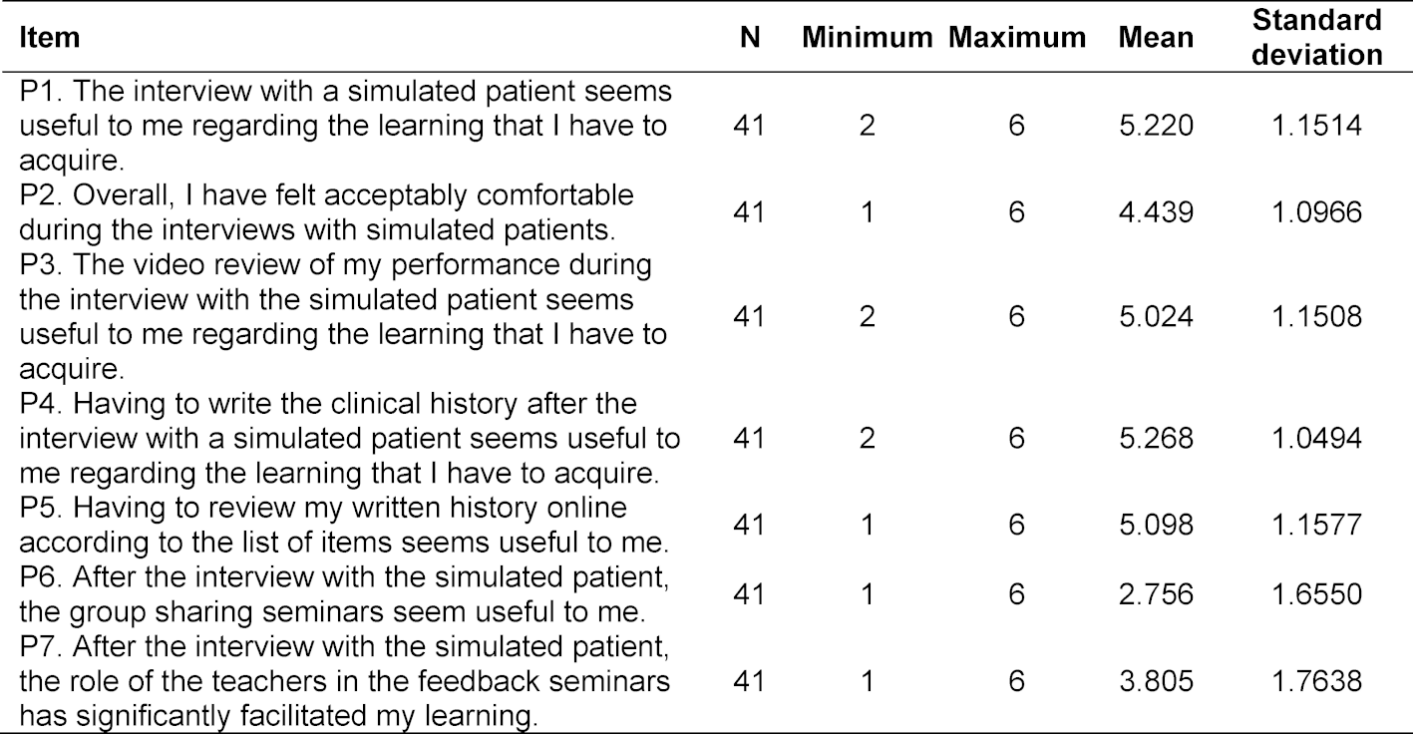
Student opinion survey results

**Table 4:**
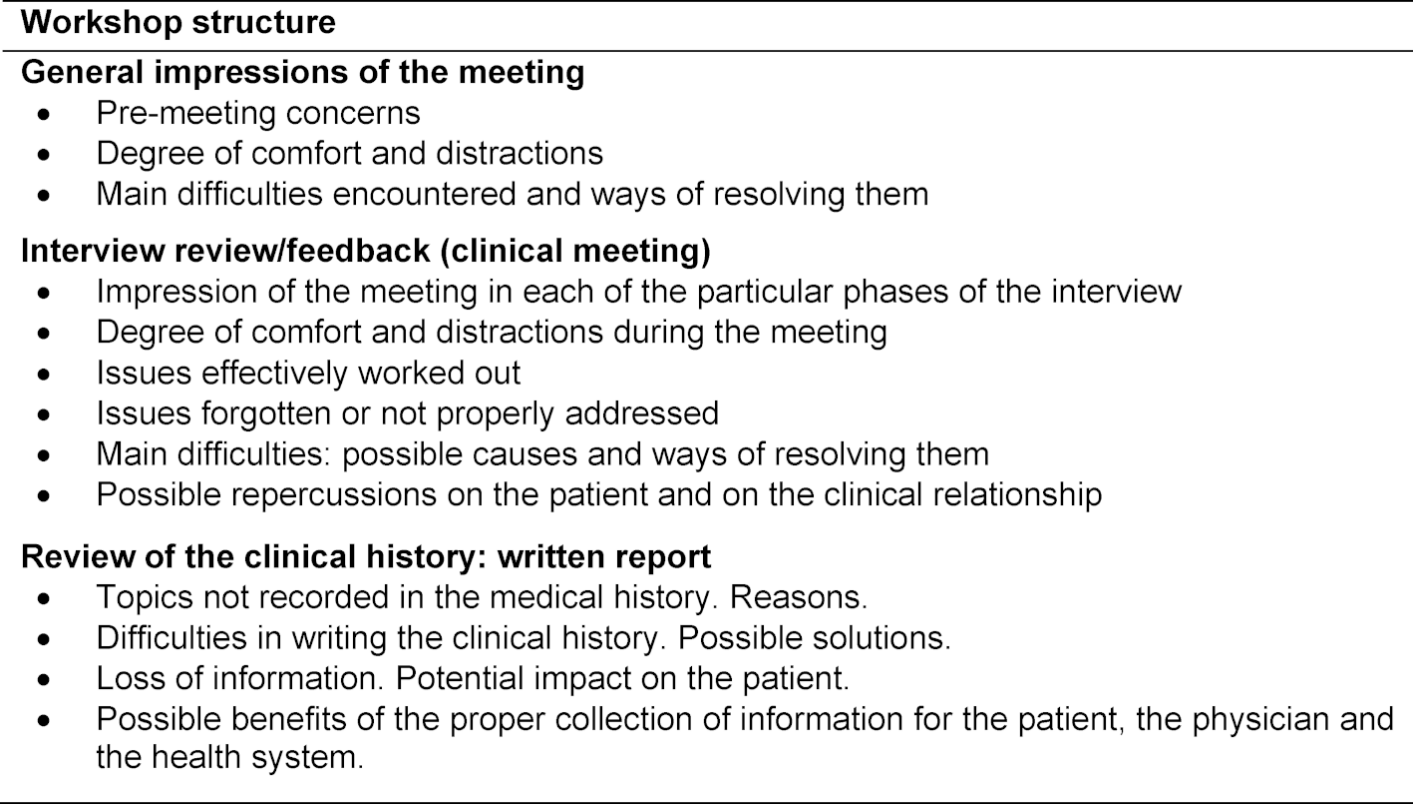
Workshop structure and topics to be addressed
